# Symptomatic Radiation Pneumonitis in NSCLC Patients Receiving EGFR-TKIs and Concurrent Once-daily Thoracic Radiotherapy: Predicting the Value of Clinical and Dose-volume Histogram Parameters

**DOI:** 10.3779/j.issn.1009-3419.2022.102.17

**Published:** 2022-06-20

**Authors:** Xuexi YANG, Ting MEI, Min YU, Youling GONG

**Affiliations:** Department of Thoracic Oncology and State Key Laboratory of Biotherapy, Cancer Center, West China Hospital, Sichuan University, Chengdu 610041, China

**Keywords:** Lung neoplasms, EGFR-TKIs, Radiation pneumonitis, Risk factor, Dose-volume histogram parameters

## Abstract

**Background and objectives:**

The incidence of symptomatic radiation pneumonitis (RP) and its relationship with dose-volume histogram (DVH) parameters in non-small cell lung cancer (NSCLC) patients receiving epidermal growth factor receptor-tyrosine kinase inhibitors (EGFR-TKIs) and concurrent once-daily thoracic radiotherapy (TRT) remain unclear. We aim to analyze the values of clinical factors and dose-volume histogram (DVH) parameters to predict the risk for symptomatic RP in these patients.

**Methods:**

Between 2011 and 2019, we retrospectively analyzed and identified 85 patients who had received EGFR-TKIs and once-daily TRT simultaneously (EGFR-TKIs group) and 129 patients who had received concurrent chemoradiotherapy (CCRT group). The symptomatic RP was recorded according to the Common Terminology Criteria for Adverse Event (CTCAE) criteria (grade 2 or above). Statistical analyses were performed using SPSS 26.0.

**Results:**

In total, the incidences of symptomatic (grade≥2) and severe RP (grade≥3) were 43.5% (37/85) and 16.5% (14/85) in EGFR-TKIs group *vs* 27.1% (35/129) and 10.1% (13/129) in CCRT group respectively. After 1:1 ratio between EGFR-TKIs group and CCRT group was matched by propensity score matching, *chi-square* test suggested that the incidence of symptomatic RP in the MATCHED EGFR-TKIs group was higher than that in the matched CCRT group (*χ*^2^=4.469, *P*=0.035). In EGFR-TKIs group, univariate and multivariate analyses indicated that the percentage of ipsilateral lung volume receiving ≥30 Gy (ilV_30_) [odds ratio (OR): 1.163, 95%CI: 1.036-1.306, *P*=0.011] and the percentage of total lung volume receiving ≥20 Gy (tlV_20_) (OR: 1.171, 95%CI: 1.031-1.330, *P*=0.015), with chronic obstructive pulmonary disease (COPD) or not (OR: 0.158, 95%CI: 0.041-0.600, *P*=0.007), were independent predictors of symptomatic RP. Compared to patients with lower ilV_30_/tlV_20_ values (ilV_30_ and tlV_20_ < cut-off point values) and without COPD, patients with higher ilV_30_/tlV_20_ values (ilV_30_ and tlV_20_ > cut-off point values) and COPD had a significantly higher risk for developing symptomatic RP, with a hazard ratio (HR) of 1.350 (95%CI: 1.190-1.531, *P* < 0.001).

**Conclusion:**

Patients receiving both EGFR-TKIs and once-daily TRT were more likely to develop symptomatic RP than patients receiving concurrent chemoradiotherapy. The ilV_30_, tlV_20_, and comorbidity of COPD may predict the risk of symptomatic RP among NSCLC patients receiving EGFR-TKIs and conventionally fractionated TRT concurrently.

## Introduction

Non-small cell lung cancer (NSCLC) is the most deadly cancer worldwide^[1]^. Targeted therapies such as epidermal growth factor receptor tyrosine kinase inhibitors (EGFR-TKIs) have greatly improved the treatment of lung cancer^[2-4]^. This type of therapy is the first choice for NSCLC patients with *EGFR* mutations due to its high selectivity and low toxicity^[5-7]^. Thoracic radiotherapy (TRT) combined with EGFR-TKIs has shown some therapeutic advantages for patients who need to receive TRT simultaneously because of lung lesions or mediastinal lymph node metastasis^[8-10]^. Presently, the National Comprehensive Cancer Network (NCCN) guidelines also recommend local treatment concurrently with the original TKIs among patients with EGFR-positive NSCLC, such as TRT^[11]^. Meanwhile, In the past, it was believed that the most important poor prognostic factor for advanced NSCLC was distant metastasis, and chemotherapy alone was the only treatment to improve survival between 2010-2015 for those patients who were diagnosed with driver-gene negative status. However, Su *et al*^[12]^. reported in Red Journal that three-dimensional radiotherapy combined with chemotherapy for primary tumor of stage Ⅳ NSCLC led to satisfactory survival outcomes with acceptable toxicity in a prospective multi-institutional phase 2 study, and some of these participants were recruited and treated in our center. When immunity therapy such as antibodies against programmed death protein 1 (PD-1) was not used for patients with metastatic NSCLC without sensitising *EGFR*/anaplastic lymphoma kinase (*ALK*) alterations, numberous prospective clinical studies^[12-14]^ reported that three-dimensional radiotherapy combined with chemotherapy for primary tumor of stage Ⅳ NSCLC has the significance of prolonging survival rates. Radiation pneumonitis (RP) is a common complication of TRT that seriously affects patients' quality of life and contributes to mortality^[15, 16]^. So far, clinical and dosimetric factors, such as age, smoking status, concurrent chemotherapy, pulmonary function, tumor location mean lung dose (MLD), gross tumor volume (GTV), V_5/10/13/20/30_ (percentage of the lung volume receiving ≥5 Gy, 10 Gy, 13 Gy, 20 Gy, 30 Gy), and heart dosimetric variables have been used to predict RP^[17-23]^. In addition, a series of data have reported that drug-induced interstitial lung disease (ILD) is seen in NSCLC patients receiving EGFR-TKIs. This is a rare but potentially life-threatening complication with a probability of occurring in the range of 0.5%-6%^[7, 24, 25]^. Very recently, Jia *et al*^[26]^. reported that the incidence and severity of RP increased in patients with TRT combined with Osimertinib, but only nine patients were included in this small study.

To date, whether the incidence of RP is increased by the routine prescription of EGFR-TKIs has not been addressed, nor has the potential predictive value of clinical and dose-volume histogram (DVH) parameters. In the present study, we reported the incidence of symptomatic RP (grade 2 or above) in NSCLC patients receiving first- and second-generation EGFR-TKIs and once-daily TRT, observed whether the incidence and intensity of symptomatic RP were further increased by comparing with patients receiving concurrent chemoradiotherapy (CCRT), and evaluated the usefulness of the clinical factors and DVH parameters for predicting the occurrence of asymptomatic RP.

## Materials and methods

### Patients

Between October, 2011 and December, 2019, we retrospectively analyzed 1, 279 patients with NSCLC who had received EGFR-TKIs and 3, 206 patients with chemotherapy at West China Hospital, Sichuan University. The inclusion criteria were as follows: the tumor stage was stage Ⅳ; once-daily conventional fractionated TRT; intensity modulated radiation therapy (IMRT) or 3-dimensional conformal radiation therapy (3D-CRT); Eastern Cooperative Oncology Group (ECOG) performance status of 0 to 1; and RP occurring during the 6 months after the completion of RT. A total radiation dose of at least 50 Gy was prescribed to the thoracic lesions, including the original tumor or metastatic lymph nodes. Finally, 214 patients were eligible for the final analysis, including 85 patients receiving EGFR-TKIs and once-daily TRT simultaneously (EGFR-TKIs group) and 129 patients receiving concurrent chemoradiotherapy (CCRT group).

### Clinical data and DVH parameters

We collected and recorded data for 17 clinical variables, including age, gender, ECOG performance status, smoking status, pathological patterns, tumor-node-metastasis (TNM) stage, tumor sites, laterality, *EGFR* mutation species, EGFR-TKIs species, presence of weight loss 6 months prior to RT, use of hormone drugs or opioids, metastatic sites, and presence of COPD. Meanwhile, we extracted and calculated 23 DVH parameters from the RT planning system incorporating the gross tumor volume (GTV), total/ipsilateral/contralateral lung V_5/10/20/30_, mean lung dose (MLD), V_10/20/30/40/50_ of heart, prescription dose, planning target volume (PTV), and total lung volume (TLV). V_x_ was defined as the percentage of lung/heart volume receiving x Gy. The lung volume was defined as the volume of the total/ipsilateral/contralateral lung minus the GTV^[27, 28]^.

### Radiotherapy

Radiotherapy was performed using once-daily IMRT/3D-CRT, and the median prescription dose was 58 Gy (range: 50 Gy-66 Gy) at 2.0 Gy per fraction. The targets were delineated based on International Commission on Radiation Units and Measurements (ICRU) reports 62^[29]^ and 83^[30]^, similar to that reported previously^[31, 32]^. The GTV was defined as an identifiable tumor including lymph nodes with a diameter of more than 1 cm on computed tomography (CT). The clinical tumor volume (CTV) included the GTV, which included 5 mm and 8 mm of surrounding lung and lymph node tissue, respectively. The PTV was created by isotropically adding a 10 mm margin to the CTV. The planning organ at risk volumes (PRVs) extended to 5 mm around the spinal cord.

The dose-volume constraints were as follows: to the total lung, V_5_ < 65%, V_20_ < 35%, and MLD < 20 Gy; and to the heart, V_30_ < 40%, V_40_ < 30%. The maximum dose allowed for the spinal cord PRV was 50 Gy. Our treatment plan system (TPS; Philips Pinnacle 3, Milpitas, USA) generated all plans, and 6-MV photon beams were delivered.

### End point definitions

The endpoint was the diagnosis of symptomatic RP, was defined as grade 2 or above RP, occurring within 6 months after the completion of TRT. severe RP was defined as grade≥3 RP, which might occur during the three months after radiotherapy, may lead to chronic complications including lung fibrosis or pulmonary failure, causing decreased life quality, treatment failure, life-threatening symptoms, and requiring oxygen support or hospitalization according to the Common Terminology Criteria for Adverse Events, version 6.0^[33]^. The diagnosis of symptomatic RP (grade 2) was confirmed by at least two experienced radiation oncologists according to clinical symptoms or changes in CT images.

### Statistical methods

First, univariate logistic regression analysis was used to evaluate the predictive value of each factor for RP (grade≥2). Second, factors with *P* < 0.05 in univariate analyses were used in multivariate analysis. *Kaplan*-*Meier* analysis was used to plot the cumulative incidence of symptomatic RP in two groups. Propensity score matching (PSM) was used to match different groups, and *chi-square* test was used to compare the incidence of symptomatic RP between the two groups. *Spearman's* rank correlation analyses were performed to prevent multicollinearity among factors. Area under the curve (AUC) of receiver operating characteristic (ROC) analysis was applied to determine the optimal cut-off value of those predictors. The *Cox* regression model was used to define the incidence curves of symptomatic RP (grade≥2) and obtain a hazard ratio (HR). Statistical analyses were performed using SPSS (version 26.0, IBM Corp, Armonk NY, USA). All tests were two-sided, and a value of *P* < 0.05 was considered statistically significant.

## Results

### Patient characteristics

The baseline characteristics of the present population are summarized in Tab 1. Most of these patients were male and had a history of smoking. Overall, 99 (46.3%) and 85 (39.7%) patients were diagnosed with N2 and N3 disease, respectively. A total of 144 (67.3%) patients had an ECOG performance status of 0. There were 43 patients with chronic obstructive pulmonary disease (COPD), accounting for 20.4% of the total population. There were 11 (12.9%) patients taking Gefitinib, 16 (18.8%) taking Erlotinib, 11 (12.9%) taking Icotinib, and 47 (55.4%) taking Afatinib in EGFR-TKIs.

**Table 1:** Baseline characteristics of all patients (*n*=214)

Baseline characteristics	Number of patients
Age (yr), Median (IQR)	58 (51-65)
Gender	
Male	128 (59.8%)
Female	86 (40.2%)
ECOG performance status	
0	144 (67.3%)
1	70(32.7%)
Pathological patterns	
Squamous carcinoma	48 (22.4%)
Adenocarcinoma	166 (77.6%)
Tumor sites	
Upper lobe	135 (63.1%)
Middle/Lower lobe	79 (36.9%)
Laterality	
Left	89 (41.6%)
Right	125 (58.4%)
Smoking status	
Yes	148 (69.2%)
No	66 (30.8%)
T stage	
T1/T2/T3/T4	16 (7.5%)/93 (43.5%)/ 40 (18.7%)/65 (30.3%)
N stage	
N0/N1/N2/N3	9 (4.2%)/21 (9.8%)/ 99 (46.3%)/85 (39.7%)
Tumor stage	
IVa/IVb	72 (33.6%)/142 (66.4%)
Therapy	
EGFR-TKIs with RT	85 (39.7%)
CCRT	129 (60.3%)
Metastatic sites	
Bone/Liver/Brain/Adrenal glands	49 (22.9%)/14 (6.5%)/ 31 (14.5%)/9 (4.2%)
COPD	
Yes/No	43 (20.1%)/171 (79.9%)
Radiation dose (Gy), Median (IQR)	50.0 (50.0-63.0)
PTV (cm^3^), Median (IQR)	238.7 (185.8-333.1)
TLV (cm^3^), Median (IQR)	2, 811 (2, 379-3, 453)
IQR: interquartile range; ECOG: Eastern Cooperative oncology Group; EGFR-TKIs: epidermal growth factor receptor tyrosine kinase inhibitors; RT: radiation therapy; CCRT: concurrent chemoradiotherapy; PTV: planning target volume; TLV: total lung volume.	

### Kaplan-Meier survival analysis

**Figure 1:**
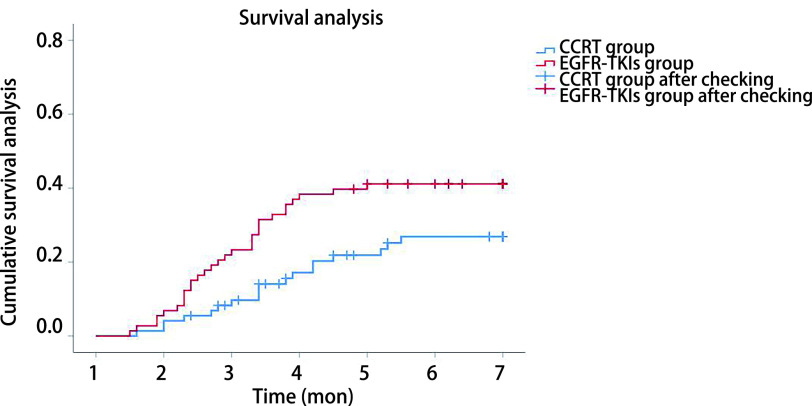
Cumulative incidence curve of symptomatic RP in EGFR-TKIs group and CCRT group

Within 6 months after radiotherapy, in total, the incidences of symptomatic RP (grade≥2) and severe RP were 33.6% (72/214) and 12.6% (27/214). The incidence of symptomatic RP (RP≥grade 2) and severe RP was 43.5% (37/85) and 16.5% (14/85) in EGFR-TKIs group vs 27.1% (35/129) and 10.1% (13/129) in CCRT group, respectively. *Kaplan-Meier* survival analysis described the cumulative incidence curve for symptomatic RP in two groups (*χ*^2^=7.309, *P*=0.007), as shown in Fig 1. Due to the small number of end point events, the median time for the occurrence of symptomatic RP in the two groups could not be calculated.

### Univariate analysis and multivariate analysis

*Logistic* regression indicated that there was no significant difference between the two groups in other baseline characteristics except pathological type (*P*≤0.001). Univariate analysis and multivariate analysis indicated that the different treatments (EGFR-TKIs/CCRT), tlV_10_(%), tlV_20_(%) and ilV_30_(%) were independent predictors of symptomatic RP in total patients.

### Propensity score matching (PSM)

These factors including pathological type, the different treatments (EGFR-TKIs/CCRT), tlV_10_(%), tlV_20_(%) and ilV_30_(%) were defined as matching variables, the callipers value was 0.02. Finally, 73 pairs were matched by PSM in two groups. According to the *chi-square* test, the incidence of symptomatic RP in the matched EGFR-TKIs group and the matched CCRT group was 41.1% (30/73) and 24.7% (18/73), respectively (*χ*^2^=4.469, *P*=0.035).

### Predictors of symptomatic RP in the EGFR-TKIs group Univariate analysis

In EGFR-TKIs group, patients with symptomatic RP were divided into group 1 (*n*=37), and the others were divided into group 2 (*n*=48). As shown in Tab 2-Tab 4, univariate analysis indicated that among clinical and pathological features, age ≤60 yr or > 60 yr [odds ratio (OR): 4.044, 95% confidence interval (CI): 3.986-4.170, *P*=0.044], with or without opioids (OR: 4.896, 95%CI: 3.481-6.284, *P*=0.027), with or without COPD (OR: 9.052, 95%CI: 8.329-10.383, *P*=0.003) demonstrated significant correlations with the incidence of symptomatic RP in the study population. There was no difference about the occurrence of RP in the patients with different types of EGFR-TKIs combined with TRT (OR: 0.607, 95%CI: 0.529-1.485, *P* > 0.05). Among the DVH parameters, tlV_10_ (OR: 1.068, 95%CI: 1.007-1.131, *P*=0.028), tlV_20_ (OR: 1.187, 95%CI: 1.075-1.311, *P*=0.001), tlV_30_ (OR: 1.248, 95%CI: 1.093-1.425, *P*=0.001), tlMD (OR: 1.003, 95%CI: 1.001-1.005, *P*=0.001), ilV_5_ (OR: 1.053, 95%CI: 1.016-1.091, *P*=0.004), ilV_10_ (OR: 1.062, 95%CI: 1.020-1.105, *P*=0.003), ilV_20_ (OR: 1.088, 95%CI: 1.032-1.146, *P*=0.002), ilV_30_ (OR: 1.0107, 95%CI: 1.044-1.173, *P*=0.001), and iMLD (OR: 1.001, 95%CI: 1.000-1.003, *P*=0.007) were significantly associated with symptomatic RP.

**Table 2:** Univariate analysis of the ability of DVH parameters to predict RP (grade≥2) in EGFR-TKIs group

DVH parameters	Symptomatic RP (*n*=37, Mean±SD)	Without RP (*n*=48, Mean±SD)	Univariate analysis OR (95%CI)	*P*
Total lungs				
V_5_ (%)	45.59±10.34	41.14±11.30	1.039 (0.997-1.083)	0.069
V_10_ (%)	34.07±7.66	29.96±8.34	1.068 (1.007-1.131)	0.028
V_20_ (%)	24.39±5.93	19.58±5.11	1.187 (1.075-1.311)	0.001
V_30_ (%)	15.22±4.51	11.79±3.75	1.248 (1.093-1.425)	0.001
MD (cGy)	1, 171.81±250.47	948.73±301.94	1.003 (1.001-1.005)	0.001
Contralateral lung				
V_5_ (%)	25.13±11.94	24.41±11.40	1.005 (0.969-1.044)	0.776
V_10_ (%)	13.91±7.73	12.24±8.87	1.024 (0.973-1.079)	0.362
V_20_ (%)	5.33±4.65	4.97±4.75	1.017 (0.928-1.115)	0.721
V_30_ (%)	1.91±2.29	2.12±2.89	0.971 (0.822-1.146)	0.725
MD (cGy)	455.75±212.57	439.31±234.03	1.000 (0.998-1.002)	0.736
Ipsilateral lung				
V_5_ (%)	66.92±13.58	57.98±12.71	1.053 (1.016-1.091)	0.004
V_10_ (%)	55.50±13.09	47.06±10.78	1.062 (1.020-1.105)	0.003
V_20_ (%)	41.08±9.20	33.77±9.52	1.088 (1.032-1.146)	0.002
V_30_ (%)	28.75±8.25	20.64±9.86	1.107 (1.044-1.173)	0.001
MD (cGy)	1, 901.73±449.09	1, 615.46±443.98	1.001 (1.000-1.003)	0.007
Heart				
V_10_ (%)	30.70±22.11	26.65±21.78	1.009 (0.989-1.029)	0.398
V_20_ (%)	19.98±16.98	15.70±17.07	1.015 (0.989-1.042)	0.257
V_30_ (%)	11.90±10.98	9.54±11.44	1.019 (0.980-1.060)	0.341
V_40_ (%)	5.60±6.31	4.68±6.67	1.022 (0.956-1.093)	0.517
V_50_ (%)	2.50±4.23	1.99±3.24	1.039 (0.923-1.169)	0.528
Radiation dose (Gy)	57.30±6.14	55.10±5.77	1.064 (0.989-1.145)	0.098
PTV (cm^3^)	276.21±88.95	243.00±85.60	1.004 (0.999-1.009)	0.087
TLV (cm^3^)	2, 818.90±753.65	2, 855.50±622.42	1.000 (0.999-1.001)	0.804
DVH: dose-volume histogram; RP: radiation pneumonitis; SD: standard deviation; 95%CI: 95% confidence interval; OR: odds ratio; V_x_: percentage of the lung volume that received more than x Gy; MD: mean dose; COPD: chronic obstructive pulmonary disease.				

**Table 3:** Univariate analysis of the ability of clinical factors to predict RP (grade≥2) in EGFR-TKIs group

Clinical factors	OR (95%CI)	*P*
Age : ≤60 yr *vs* 60 yr	4.044 (3.986-4.170)	0.044
Gender: male *vs* female	0.001 (0.000-0.006)	0.975
ECOG: PS 1 *vs* 0	1.413 (0.596-3.478)	0.503
Squamous carcinoma *vs* adenocarcinoma	1.279 (0.541-3.020)	0.575
Upper lobe *vs* middle/lower lobe	0.176 (0.127-1.354)	0.675
Laterality: left *vs* right	0.346 (0.275-0.582)	0.556
EGFR species	0.607 (0.529-1.485)	0.078
Weight loss 6 months prior to RT: yes *vs* no	0.020 (0.017-2.842)	0.887
Hormone drugs: yes *vs* no	0.607 (0.529-1.485)	0.436
Opioids: yes *vs* no	4.896 (3.481-6.284)	0.027
Smoking status: yes *vs* no	0.981 (0.637-3.591)	0.322
T stage: T3/T4 *vs* T1/T2	1.287(1.173-3.852)	0.257
N stage: N2/N3 *vs* N0/N1	0.092(0.032-1.395)	0.761
Metastatic sites: Bone	1.656 (1.434-7.147)	0.198
Metastatic sites: Liver	0.396 (0.262-1.234)	0.529
Metastatic sites: Brian	1.264 (1.028-6.328)	0.261
Metastatic sites: Adrenal gland	0.071 (0.007-0.298)	1.000
COPD: yes *vs* no	9.052 (8.329-10.383)	0.003

**Table 4:** Multivariate analysis and ROC analysis of the ability of clinical factors and DVH parameters to predict RP (grade≥2) in EGFR-TKIs group

Factor	Multivariate analysis			ROC curve			Sensitivity	Specificity
	Regression coefficient	OR(95%CI)	*P*	AUC(95%CI)	Cut-off point	*P*		
tlV_20_	0.158	1.171 (1.031-1.330)	0.015	0.731 (0.622-0.841)	22.10%	< 0.001	0.703	0.729
ilV_30_	0.151	1.163 (1.036-1.306)	0.011	0.747 (0.615-0.878)	25.78%	< 0.001	0.757	0.729
COPD	-1.848	0.158 (0.041-0.600)	0.007	0.637 (0.515-0.759)	-	0.031	0.378	0.896
Combination of tlV_20_/ilV_30_/COPD	-	-	-	0.823 (0.734-0.912)	-	< 0.001	0.757	0.792
ROC curve: receiver operating characteristic curve; AUC: area under the curve; ilV_30_: percentage of ipsilateral lung volume receiving≥30 Gy; tlV_20_: percentage of the total lung volume receiving ≥20 Gy.								

### Multivariate analysis

As shown in Tab 5, *Spearman's* correlation analysis demonstrated relationships between the statistically significant DVH parameters. Multivariate *Logistic* regression was performed using the significant factors obtained during univariate analysis: ilV_30_ (OR: 1.163, 95%CI: 1.036-1.306, *P*=0.011), tlV_20_ (OR: 1.171, 95%CI: 1.031-1.330, *P*=0.015), and with or without COPD (OR: 0.158, 95%CI: 0.041-0.600, *P*=0.007) were independent predictive factors for symptomatic RP in the present cohort.

**Table 5:** *Spearman's* rank correlation analyses among the statistically significant DVH parameters

DVH	tlV_10_	tlV_20_	tlV_30_	tlMD	ilV_5_	ilV_10_	ilV_20_	ilV_30_	ilMD
tlV_10_	1.000	0.818	0.647	0.757	0.744	0.720	0.614	0.502	0.617
tlV_20_	0.818	1.000	0.753	0.710	0.619	0.587	0.590	0.540	0.628
tlV_30_	0.647	0.753	1.000	0.756	0.591	0.611	0.651	0.742	0.771
tlMD	0.757	0.710	0.756	1.000	0.721	0.730	0.695	0.663	0.695
ilV_5_	0.744	0.619	0.591	0.721	1.000	0.958	0.859	0.695	0.750
ilV_10_	0.720	0.587	0.611	0.730	0.958	1.000	0.907	0.743	0.815
ilV_20_	0.614	0.590	0.651	0.695	0.859	0.907	1.000	0.860	0.837
ilV_30_	0.502	0.540	0.742	0.663	0.695	0.743	0.860	1.000	0.833
ilMD	0.617	0.628	0.771	0.695	0.750	0.815	0.837	0.833	1.000
V_x_ was defined as the percentage of lung/heart volume receiving x Gy. t/i/clV_x_: total/ipsilateral/contralateral lung volume.									

### ROC curve analysis

**Figure 2:**
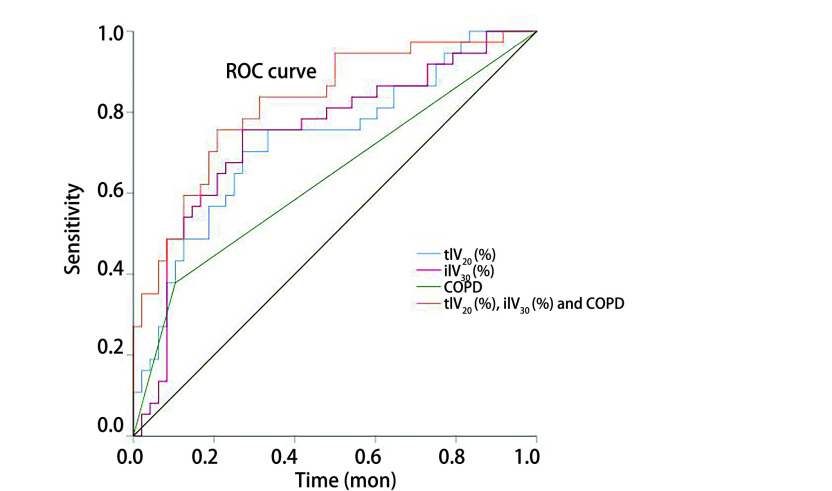
ROC curves of tlV_20_, ilV_30_, COPD, combination of tlV_20_, ilV_30_ and COPD, for symptomatic RP in the present study

The ROC curves of ilV_30_, tlV_20_, and the morbidity of COPD are shown in Fig 2. The ROC curves demonstrate that the AUC of tlV_20_ was 0.731 (95%CI: 0.622-0.841, *P* < 0.001), and its optimal cut-off point was 22.1% (sensitivity and specificity of 0.703 and 0.729, respectively). The AUC of ilV_30_ was 0.747 (95%CI: 0.615-0.878, *P* < 0.001), with an optimal cut-off point of 25.8% (sensitivity and specificity of 0.757 and 0.729, respectively). The AUC of the morbidity of COPD was 0.637 (95%CI: 0.515-0.759, *P*=0.031), with a sensitivity and specificity of 0.378 and 0.896, respectively. In the combined analysis of ilV_30_, tlV_20_, and the morbidity of COPD, the AUC was as high as 0.823 (95%CI: 0.734-0.912, *P* < 0.001), with a sensitivity and specificity of 0.775 and 0.792, respectively.

### Cox regression analysis

**Figure 3:**
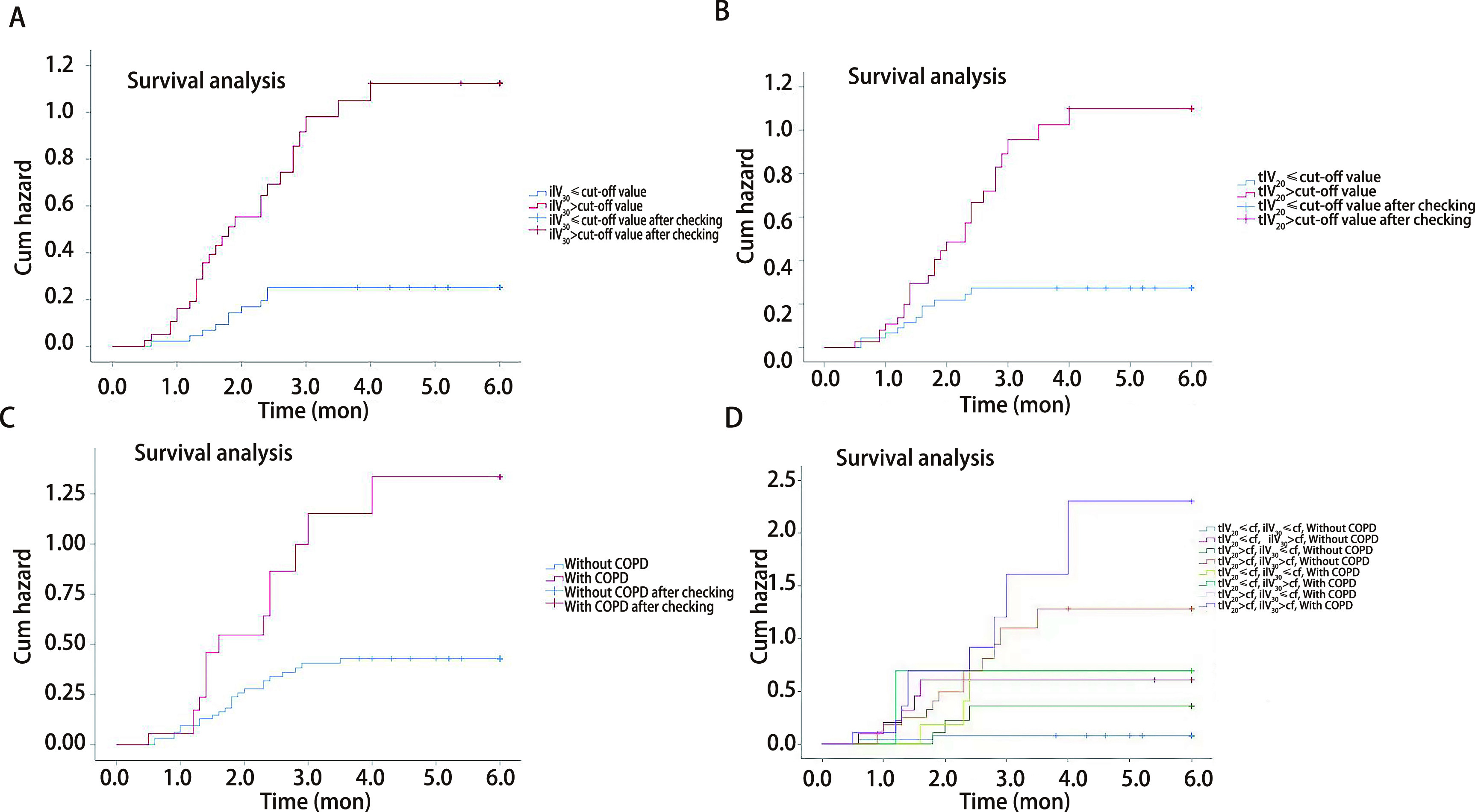
*Kaplan-Meier* estimates of cumulative hazards for symptomatic RP in the present study. A: ilV_30_-low group vs ilV_30_-high group; B: tlV_20_-low group vs tlV_20_-high group; C: with COPD *vs* without COPD; D: combination of tlV_20_, ilV_30_, and COPD.

The patients were categorized into different groups based on the cut-off point values of tlV_20_ and ilV_30_, with or without COPD. Patients in the ilV_30_-low group (ilV_30_≤cut-off point value) and patients in the ilV_30_-high group (ilV_30_ > cut-off point value) had a significantly higher risk of symptomatic RP with an HR of 4.787 (95%CI: 2.252-10.177, *P* < 0.001) (Fig 3A). The incidences of symptomatic RP in the patients in the ilV_20_-high (ilV_20_ > cut-off point value) and COPD group (patients with COPD) were significantly higher than those in the ilV_20_-low group (ilV_20_≤cut-off point value) and the non-COPD group (patients without COPD), respectively. The HRs were 3.453 (95%CI: 1.701-7.011, P≤0.001, Fig 3B) and 0.367 (95%CI: 0.188-0.716, *P* < 0.001, Fig 3C). Compared to the patients in the ilV_30_-low/tlV_20_-low/non-COPD group, patients in the ilV_30_-high/tlV_20_-high/COPD group had the highest risk of symptomatic RP in the present population, with an HR of 1.350 (95%CI: 1.190-1.531, *P* < 0.001, Fig 3D).

## Discussion

Few studies have assessed possible predictors of the risk of symptomatic RP among patients with NSCLC who had received EGFR-TKIs and once-daily TRT. To the best of our knowledge, the present study has the largest sample size of similar studies and we verified potential predictors. Our findings not only indicate that compared with CCRT, patients with EGFR-TKIs combined with TRT were more likely to develop symptomatic RP, but also identified that ilV_30_, tlV_20_, and presence of COPD had potential predictive values for the occurrence of symptomatic RP in this selected population, and the combination of these three factors was found to be meaningful.

Experimental studies have revealed the molecular mechanisms underlying the development of ILD introduced by EGFR-TKIs. Takeyama *et al*^[34]^. reported that goblet cell proliferation is an important pathological feature of airway secretory disease, and that the expression of EGFR promotes its production and evolution. Ren and colleagues^[24, 25]^ observed that improper regeneration of continuously damaged epithelial cells is an important process leading to pulmonary fibrosis. Epithelial expression of EGFR increased in fibrotic lung tissue compared with normal lung tissue, suggesting that EGFR-mediated signaling pathways are involved in epithelial regeneration of fibrotic lung disease. Moreover, an *in vivo* study by Sun *et al*^[35]^. showed that EGFR-TKIs increased inflammatory cell infiltration and produced more pro-inflammatory cytokines (IL-6 and IL-1), which stimulated the inflammatory response. Various case reports and studies continue to show significant variability in the incidence of ILD by EGFR-TKIs^[6, 7, 36-39]^. Cohen *et al*^[37]^. reviewed a safety information database containing more than 50, 000 patients treated with gefitinib worldwide and found 408 patients who had ILD, 324 of whom were from Japan. Mok *et al*^[6, 7]^. reported that approximately 4% of patients developed ILD in response to Osimertinib. Smaller studies conducted in Asia have reported higher incidences, ranging from 4%-6%^[38, 39]^.

Meanwhile, the combination of EGFR-TKIs and radiation might have a superposed effect on the pulmonary interstitium^[40-43]^. *In vivo*, EGFR-TKIs can inhibit proliferation of alveolar epithelial cells and prevent them from repairing themselves in the case of radiation damage^[44]^. In addition, EGFR-TKIs might reduce the G_2_/M phase retardation of irradiated cells and delay DNA damage repair, and are considered radiation sensitizers^[40]^. In addition, Li *et al*^[41, 42]^. reported that radiation sensitization of EGFR-TKIs increases radiation damage to normal lung tissue. From this point of view, concurrent TRT might increase the RP incidence and severity on the routine prescription of EGFR-TKIs among NSCLC patients.

The reported RP incidence range for concurrent chemo-radiotherapy is 15%-40% (symptomatic or grade≥2) and 10%-20% (severe or grade≥3), respectively^[43, 45]^. This is consistent with the incidence of symptomatic RP observed in the CCRT group in our study, but we aimed to explore the incidence of symptomatic RP in patients with EGFR-TKIs and TRT, as well as its predictors. In clinical practice, the incidence of RP in patients treated with a combination of TKIs and TRT has been observed and reported by a few researchers. Zhuang *et al*^[46]^. reported the incidence of RP in NSCLC patients treated with concurrent TRT combined with erlotinib. Among the 24 patients, nine patients (37.5%) had RP of grade 2 or above, and three patients died of RP. In their reports, the median irradiation dose and PTV volume were 57 Gy (2 Gy per fraction) and 279.70 cm^3^, respectively. Xu *et al*^[47]^. also reported that 7.7% of patients developed grade 3 or worse RP and accepted definitive radiotherapy. The EGFR-TKIs in their study included standard-fractionation radiotherapy (60 Gy in 2 Gy per fraction) and stereotactic radiosurgery (SRS) (21 Gy to 27 Gy in single fraction, 26.5 Gy to 33.0 Gy in 3 fractions, and 30 Gy to 37.5 Gy in 5 fractions). Wang *et al*^[48]^. concluded that there was a lower incidence of RP among patients receiving erlotinib combined with TRT. However, the results may be associated with lower lung exposures as the mean MLD and lung V_20_ were 8.6 Gy and 14%, respectively. Nanda *et al*^[49]^. and Chang *et al*^[50]^. reported high incidences of RP in patients receiving combined erlotinib or gefitinib combined with TRT. All of these studies had relatively small sample sizes and the predictive value of corresponding parameters was not evaluated. In the present study, we reported 43.5% grade 2 or worse RP in patients treated with combination first- and second-generation EGFR-TKIs and TRT, and 16.5% of patients developed grade 3 or worse RP. These results are similar to those mentioned above^[46, 50, 51]^, indicating that clinicians should pay close attention to the relatively higher incidence of RP if patients receive EGFR-TKIs and conventionally fractionated and high-dose TRT concurrently.

Very recently, Jia *et al*^[26]^. reported that in patients receiving third-generation Osimertinib combined with TRT, seven (7/11, 63.6%) were recorded with grade 2 or higher RP, and the incidence of severe RP was 54.5% (6/11). The authors concluded that Osimertinib and simultaneous TRT have potential lethality in some highly sensitive patients, even at low radiation doses for the organ at risk.

In our study, multivariate analysis indicated that ilV_30_ (cut-off value: 25.8%) and tlV_20_ (cut-off value: 22.1%) were independent predictive factors for symptomatic RP, from amongst all the DVH parameters. Cox regression analysis indicated that the predictive value of the combination of ilV_30_, tlV_20_, and morbidity of COPD was as high as 0.823. These results were consistent with those of previous studies. Many studies have stated that tlV_20_ is associated with the occurrence of symptomatic RP^[16, 22, 51, 52]^, Kong^[16]^ and colleagues pointed out that the cut-off point value of tlV_20_ to predict RP is 30%. Graham *et al*^[51]^. also reported that tlV_20_ could predict RP when tlV_20_ was less than 22%, there was no pneumonitis in this study. Tsujino *et al*^[52]^ reported that 51% of patients with symptomatic RP had a tlV_20_ of 26%-30%. Zhang *et al*^[18]^ reported that tlV_20_ (≥25 %) could predict symptomatic RP. In the present study, we reported a lower value of tlV_20_ and reminded physicians to be cautious when combining TRT and EGFR-TKIs. Meanwhile, several studies have shown that COPD is a useful predictor of RP^[18, 53, 54]^. Moreno *et al*^[54]^ researched 80 cases of NSCLC, and multivariate analysis showed that COPD was an independent risk factor for radiation pneumonia (*P*=0.01). COPD is closely related to chronic bronchitis and emphysema. In patients with COPD, there is a variety of inflammatory cell infiltration in the bronchiac wall, and proliferation of granulation tissue and mechanized fibrous tissue in the base, which are more likely to lead to the occurrence of RP. However, few studies have reported whether DVH parameters in the ipsilateral lung can predict RP. Dang *et al*^[55]^ reported that univariate analysis showed that V_5_-V_50_ of both the ipsilateral and total lung were related to the occurrence of RP, but failed to report the results of the DVH parameters in the ipsilateral lung in multivariate analysis. Our findings are the first to report that ilV_30_ can predict symptomatic RP in patients receiving EGFR-TKIs and TRT. When ilV_30_ is more than 25.8%, the incidence of symptomatic RP is significantly increased.

The limitations of the present study should be critically addressed. First, this was a retrospective single-center descriptive analysis, and is therefore subject to bias from multiple sources. Second, the sample size was relatively small and insufficient for obtaining a definitive conclusion. Therefore, the risk factors identified in the present study should be cautiously generalized for routine use and require validation in another independent data set. We could not compare the occurrence of RP with different types of TKIs combined with TRT and identified the respective predictors. Moreover, we only collected the data of first- and second-generation TKIs, and did not analyze the data regarding Osimertinib, which in previous studies resulted in a high incidence of RP. In particular, all patients in this cohort had received a prescription dose above 50 Gy, which could have led to an increased risk of RP. Therefore, re-simulation and plan modifications may be required in practice for patients with NSCLC.

In summary, for the first time, we report that ilV_30_, tlV_20_, and diagnosed COPD may predict the risk of symptomatic RP among NSCLC patients receiving EGFR-TKIs and conventionally fractionated TRT concurrently. These findings are relevant for radiation therapists and clinicians. It is important to note that in patients diagnosed with COPD and receiving EGFR-TKIs at the same time, caution must be paid when formulating radiotherapy planning and DVH parameters should be reduced. Studies of larger samples may identify further potential dosimetric parameters to predict RP in such patients. Meanwhile, prospective studies are needed to verify our findings.
